# From the PnTx2-6 Toxin to the PnPP-19 Engineered Peptide: Therapeutic Potential in Erectile Dysfunction, Nociception, and Glaucoma

**DOI:** 10.3389/fmolb.2022.831823

**Published:** 2022-04-11

**Authors:** Carolina Nunes da Silva, Kenia Pedrosa Nunes, Lays Fernanda Nunes Dourado, Thayllon Oliveira Vieira, Xavier Maia Mariano, Armando da Silva Cunha Junior, Maria Elena de Lima

**Affiliations:** ^1^ Departmentamento de Bioquímica e Imunologia, Universidade Federal de Minas Gerais, Belo Horizonte, Brazil; ^2^ Faculdade de Farmácia, Universidade Federal de Minas Gerais, Belo Horizonte, Brazil; ^3^ Department of Biomedical and Chemical Engineering and Sciences, Florida Institute of Technology, Melbourne, FL, United States; ^4^ Programa de Pós-Graduação em Medicina e Biomedicina Faculdade Santa Casa de Belo Horizonte, Belo Horizonte, Brazil

**Keywords:** PnTx2-6, PnPP-19, erectile dysfunction, glaucoma, pain

## Abstract

The venom of the “armed” spider *Phoneutria nigriventer* comprises several potent toxins. One of the most toxic components from this venom is the neurotoxin PnTx2-6 (LD_50_ = ∼ 0.7 μg/mouse, 48 residues, five disulfide bridges, MW = 5,289.31 Da), which slows down the inactivation of various Na^+^ channels. In mice and rats, this toxin causes priapism, an involuntary and painful erection, similar to what is observed in humans bitten by *P. nigriventer*. While not completely elucidated, it is clear that PnTx2-6 potentiates erectile function *via* NO/cGMP signaling, but it has many off-target effects. Seeking to obtain a simpler and less toxic molecule able to retain the pharmacological properties of this toxin, we designed and synthesized the peptide PnPP-19 (19 residues, MW = 2,485.6 Da), representing a discontinuous epitope of PnTx2-6. This synthetic peptide also potentiates erectile function *via* NO/cGMP, but it does not target Na^+^ channels, and therefore, it displays nontoxic properties in animals even at high doses. PnPP-19 effectively potentiates erectile function not only after subcutaneous or intravenous administration but also following topical application. Surprisingly, PnPP-19 showed central and peripheral antinociceptive activity involving the opioid and cannabinoid systems, suggesting applicability in nociception. Furthermore, considering that PnPP-19 increases NO availability in the corpus cavernosum, this peptide was also tested in a model of induced intraocular hypertension, characterized by low NO levels, and it showed promising results by decreasing the intraocular pressure which prevents retinal damage. Herein, we discuss how was engineered this smaller active non-toxic peptide with promising results in the treatment of erectile dysfunction, nociception, and glaucoma from the noxious PnTx2-6, as well as the pitfalls of this ongoing journey.

## Introduction

While animal venoms are a real treasure because of their biodiversity, they also represent a great challenge to be deciphered by scientists. Brazil holds more than 20% of the world’s biodiversity, which embodies a rich source of natural components for research with huge pharmacological potential (De Lima et al., 2010; De Marco Almeida et al., 2015). Animal venoms are complex mixtures of salts, small molecules, such as amino acids, biogenic amines, neurotransmitters, peptides, and proteins, and therefore, they show a multitude of combinatorial active components. Overall, these venoms and their toxins have a wide range of pharmacological activities that are relevant tools to study different biological functions at the cellular and molecular level. Some molecules in the venom play important roles in human diseases and have been used to design new therapeutic agents (Bordon et al., 2020). Noteworthy, for over half a century, Sergio Ferreira (Ferreira, 1965) studied the snake venom of *Bothrops jararaca* in Brazil. He discovered some peptides—BPFs (bradykinin potentiator factors), which potentiate bradykinin, a vasodilator that reduces blood pressure by inhibiting the angiotensin II converting enzyme (ACE). From Ferreira’s work, captopril emerged as one of the most used antihypertensive drugs worldwide. Two other examples of FDA-approved peptides derived from animal venoms are: 1) Ziconotide or Prialt^®^—analgesic drug originally purified as ω-conotoxin MVIIa peptide from a marine cone snail (*Conus magus*), which was discovery by Professor Baldomero Olivera’s group; and 2) Exenatide—an anti-diabetic synthetic peptide (Byetta^®^), from Exendin-4, a hormone found in the saliva of the Gila monster (*Heloderma suspectum*). Several other peptides in clinical and preclinical trials (for review, please see Herzig et al., 2020), illustrates the translational power of venoms. An estimated 20 million different compounds can only be found in venom from spiders. (King and Hardy, 2013).

Here we describe the activity of the toxin PnTx2-6 or δ-CNTX-Pn2a (following the nomenclature suggested by King et al. (2008), a highly toxic peptide obtained from the venom of the spider *Phoneutria nigriventer*. Searching for understanding its mechanism leading to priapism and seeking to eliminate its toxicity, the smaller peptide PnPP-19 (*P. nigriventer* potentiator peptide) was engineered by using PnTx2-6 molecule as a template (deposited in UniProtKB - P29425). This peptide showed great potential as a therapeutic drug without significant toxicity. Surprisingly, PnPP-19, initially investigated to treat erectile dysfunction (ED), also showed relevant antinociceptive action besides efficacy against glaucoma in rodent models. In this review, we discuss PnTx2-6 and PnPP-19 peculiarities and how this peptide emerged as a potential drug to treat ED, nociception, and glaucoma. We provide an overview of about how PnPP-19 can target these pathological conditions, highlighting a parallel between the toxin and this peptide.

## The Toxin PnTx2-6 *Versus* PnPP-19 in Erectile Dysfunction

### The Erectile Mechanism

The erectile mechanism is the result of an intricate neurovascular process that comprises a sensitive machinery, encompassing multiple pathways, in which the most important mediator is still nitric oxide (NO). Penile erection can be initiated from peripheral stimuli or the central nervous system (CNS). The molecular basis behind the signal processing is extensively studied (for review, please see (Andersson, 2011), but not yet completely understood. Sexual arousal in males is found predominantly in the limbic system and hypothalamus and stimulation in these areas are associated with erection in rats. Many neuronal circuits and pathways are involved in the entire process. On the other hand, the local mechanisms leading to penile erection requires the relaxation of smooth muscle cells in the corpus cavernosum (CC), which is triggered by releasing NO from neuronal NO synthase (nNOS) in nitrergic nerves and from endothelial (e)NOS. NO diffuses into adjacent muscle cells, binding to soluble guanylyl cyclase (sGC) catalyzing the conversion of guanosine triphosphate (GTP) to cyclic guanosine monophosphate (cGMP), a mechanism called the NO/cGMP pathway, which decreases cytosolic Ca^2+^, leading to the tumescence stage (Nunes and Webb, 2012).

For detumescence to occur, the process needs to invert towards vasoconstriction, which involves the hydrolysis of cGMP by phosphodiesterase type 5 (PDE5) and activation of the Rho-kinase pathway. Any imbalance in these pathways, might cause ED, defined as a persistent inability to achieve or maintain a satisfactory penile erection (NIH, 1993). Undoubtedly, the oral selective PDE-5 inhibitors are the standard of care for treating ED. However, because their action requires intact nitrergic nerve fibers, these drugs showed limitations and are not the best solution when NO production is impaired. PDE-5 inhibitor therapy fails in approximately 30–40% of men with ED (Goldstein et al., 2012; Porst et al., 2013; Simonsen et al., 2016). In addition, although present research in this field is investigating cutting-edge therapeutic strategies, such as gene and cell-based technologies aiming to find a cure for ED, much research still needs to be done and it is unknown if these resources encompass all types of patients. Hence, a demand for novel low-cost approaches is evident, especially if they are efficient for treating ED in hypertensive, diabetic, and older patients.

### PnTx2-6: The Link Between the *Phoneutria nigriventer* Venom and Erection

Priapism is defined as persistent tumescence unrelated to sexual stimulation, and it is a medical emergency caused by decreased or absent venous drainage. This condition is pointed out as a clinical manifestation following systemic intoxication caused by biting of *P. nigriventer* spider in humans and animals. Accordingly, it was reasonable to assume the presence of toxins in this spider venom triggering an erection, with worthwhile implications. While animal venoms are a miscellaneous of bioactive components with broad application following purification and isolation (for review, please see (De Lima et al., 2010; Rates et al., 2011; Peigneur et al., 2018; Bordon et al., 2020), unfortunately, it is very challenging and time consuming to extract animal’s venom. Additionally, it is expensive and hard to obtain a significant amount of purified toxins to perform reliable experiments. To address these limitations, researchers identified, isolated, and synthesized peptides of utmost importance from the venom of *P. nigriventer* (PV) without compromising their biological potential, while improving their pharmacological properties.

**FIGURE 1:**
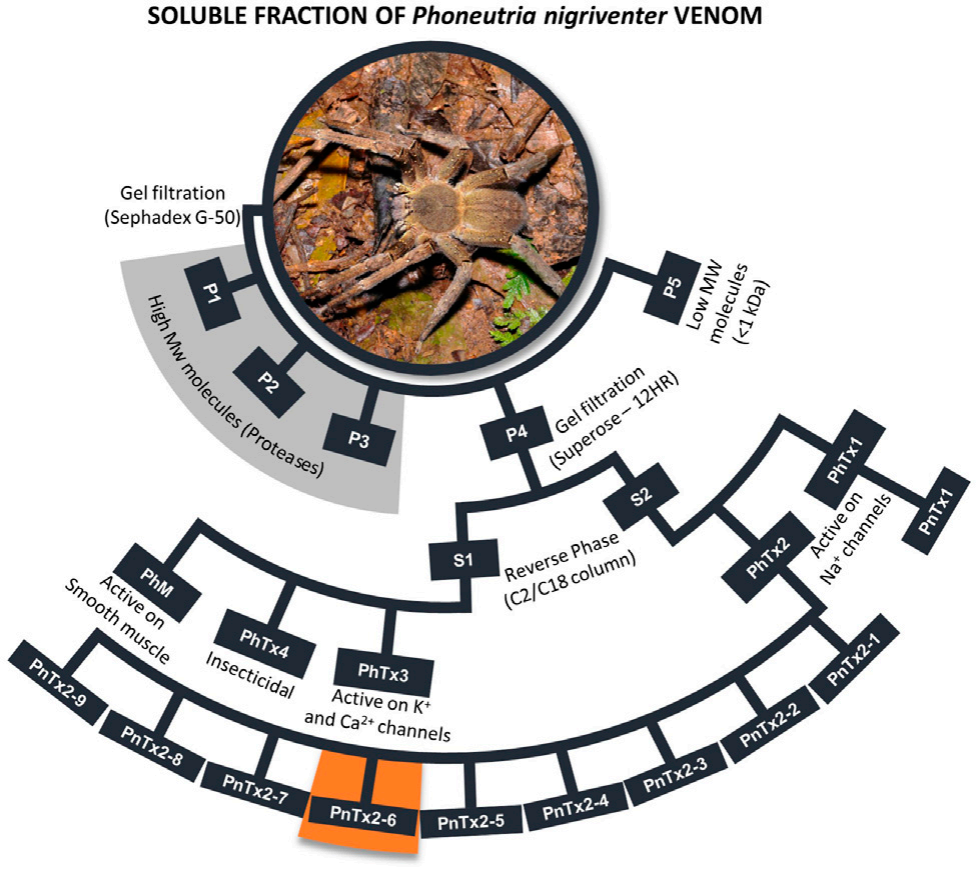
Scheme of purification *P. nigriventer* spider venom summarizing the general activities of each fraction. All toxins obtained from fraction PhTx2 were showed, including PnTx2-6 highlighted in orange. More detailed studies can be found in Peigneur et al. (2018). The names of the toxins are presented as in the first purification of the venom. The nomenclature suggested by King et al. (2008) for these toxins can be found in the Arachnoserver database (https://www.uniprot.org/database/DB-01450), King et al., 2008 and Peigneur et al., 2018. Photo credit: R. B. Aronson, Florida Tech (United States).

The purification of this venom (for review, please see De Lima et al., 2010; Peigneur et al., 2018) generated five semi purified fractions (Figure 1), and each of them was subsequently purified generating several toxins, which are active in ionic channels, mainly sodium (Nav) and calcium (Cav) channels, except the fraction PhTx1, which only includes one toxin (PnTx1) active in Nav. Some of these toxins and their targets were not clarified yet. Figure 1 highlights the nine toxins obtained from the PhTx2 fraction, and two of them, PnTx2-5 (δ-ctenitoxin-Pn2c) and PnTx2-6 (formerly Tx2-5 and Tx2-6), are associated to the dominant excitatory symptoms of the venom, including priapism. Both toxins have 48 residues, share a 90% sequence similarity, and are reticulated by five disulfide bridges (Cordeiro et al., 1992; Matavel et al., 2009). The first pharmacological study showed that the semi purified fraction PhTx2 prolonged the inactivation and deactivation of Na^+^ channel currents (Araújo et al., 1993). The toxin PnTx2-5, intraperitoneally injected in mouse, induced a toxic syndrome, characterized by priapism, hypersalivation, pulmonary edema, and death by respiratory distress. Some of these effects were reduced, while priapism and others were prevented by a pretreatment with NOS inhibitors (Yonamine et al., 2004), suggesting a NO-dependent effect caused by PnTx2-5. Interestingly, microarray analysis in mice CC inoculated with PnTx2-6 showed that 10.4% of the genes involved in the NO pathway were differentially expressed (Villanova et al., 2009).

In the first toxicity test, mice were intracerebroventricularly injected with PnTx2-6 and scratching, lachrymation, hypersalivation, agitation, spastic paralysis of anterior and posterior extremities at 0.79 μg/mouse dosage was observed (Cordeiro et al., 1992). Electrophysiological experiments in GH3 cells, showed that PnTx2-6 delays the fast inactivation kinetics of neuronal-type Na^+^ channels. Combined with binding experiments, it was suggested that the mode of action of this toxin is more like those toxins acting at site 3 of Na^+^ channels, although it also showed some similarities with ß-toxins (Matavel et al., 2009). Site 3 is known to be targeted by α-toxins, whereas site 4 is targeted by ß-toxins of scorpion. Ionic channels are crucial regulators of cell excitability and a large number of toxins act on them modifying their properties, as showed in numerous papers (Catterall et al., 2007), which explains why they directly or indirectly trigger many changes in cell physiology. Our group demonstrated that PnTx2-6 potentiates erectile function in many disease models (Nunes et al., 2008, 2010; 2012a). However, high toxicity and nociception were observed (Nunes, 2008). Further investigations become restricted because the concentration of this toxin in the venom is very small. Therefore, to overcome this drawback, a functional protein with similar activity in erectile function as the native toxin, was obtained by heterologous expression in *E. coli* (Torres et al., 2010). However, the toxicity was yet a big challenge before reaching further steps to make this protein a possible pharmacological intervention.

Seeking minimize toxicity, PnPP-19 (*Phoneutria nigriventer* potentiator peptide with 19 amino acid residues) was designed based on the theoretical structure of the toxin, by using a bioinformatical approach, which selected the most exposed region of the toxin as its probable interaction site with Na^+^ channel (Fleury, 2009; Matavel et al., 2009). Therefore, PnPP-19 represents some discontinuous epitopes of PnTx2-6. In addition, to provide higher stability to the molecule, it was carboxylated at N-terminal, amidated at C-terminal, and the Cys residue in the C-terminal was replaced by a Ser (Table 1). Structurally, the linear peptide PnPP-19 may present an α-helical motif that is in agreement with the proposal for the active core of PnTx2-6 (Moreau et al., 2008; Matavel et al., 2009; Silva et al., 2015).

**TABLE 1:** Pharmacological, biochemical, and physiological data comparing the toxin PnTx2-6 and the synthetic peptide PnPP-19.

	PnTx2-6 Native Toxin	PnPP-19 Synthetic peptide	References
MW and (# of aa) Obtention	5,289.31 Da (48) Purification from the venom or heterologous expression	2,485.6Da (19) Chemically synthesized	(Cordeiro et al., 1992; Matavel et al., 2009; Silva et al., 2015)
Toxicity (LD50)	0.79 µg/mouse (0.032 mg/kg)	Not detected until 5 mg/kg in mouse	(Cordeiro et al., 1992; Silva et al., 2015)
Activity on ionic channels	Different voltage-gated Na^+^ channel (Na_v_) subtypes	Not detected	Silva et al. (2015)
Primary structure comparation	ATCAGQDQPCKETCDCCGERGECVCGGPCICRQGYFWIAWYKLANCKK	Ac-GERRQYFWIAWYKLANSKK-NH_2_	(Matavel et al., 2009; Silva et al., 2015)
Relaxation of CC in vivo	++++	++++	(Nunes et al., 2008; Silva et al., 2015)
Effect on nociception	Nociceptive	Antinociceptive	(Nunes, 2008; Da Fonseca Pacheco et al., 2016; Freitas et al., 2016)
Relaxation of CC in vitro	++++	++++	(Nunes et al., 2010; Silva et al., 2015)
Priapism	++++	**- - - -**	Cordeiro et al. (1992)
Immunogenicity	ND	Very low	Silva et al. (2015)

# of aa, number of aminoacids; (++++) indicates presence; (**- - - -**) indicates absence; ND, not determined; CC, corpus cavernosum.

#### Pro-erectile Action of PnTx2-6 *Versus* PnPP-19

**FIGURE 2:**
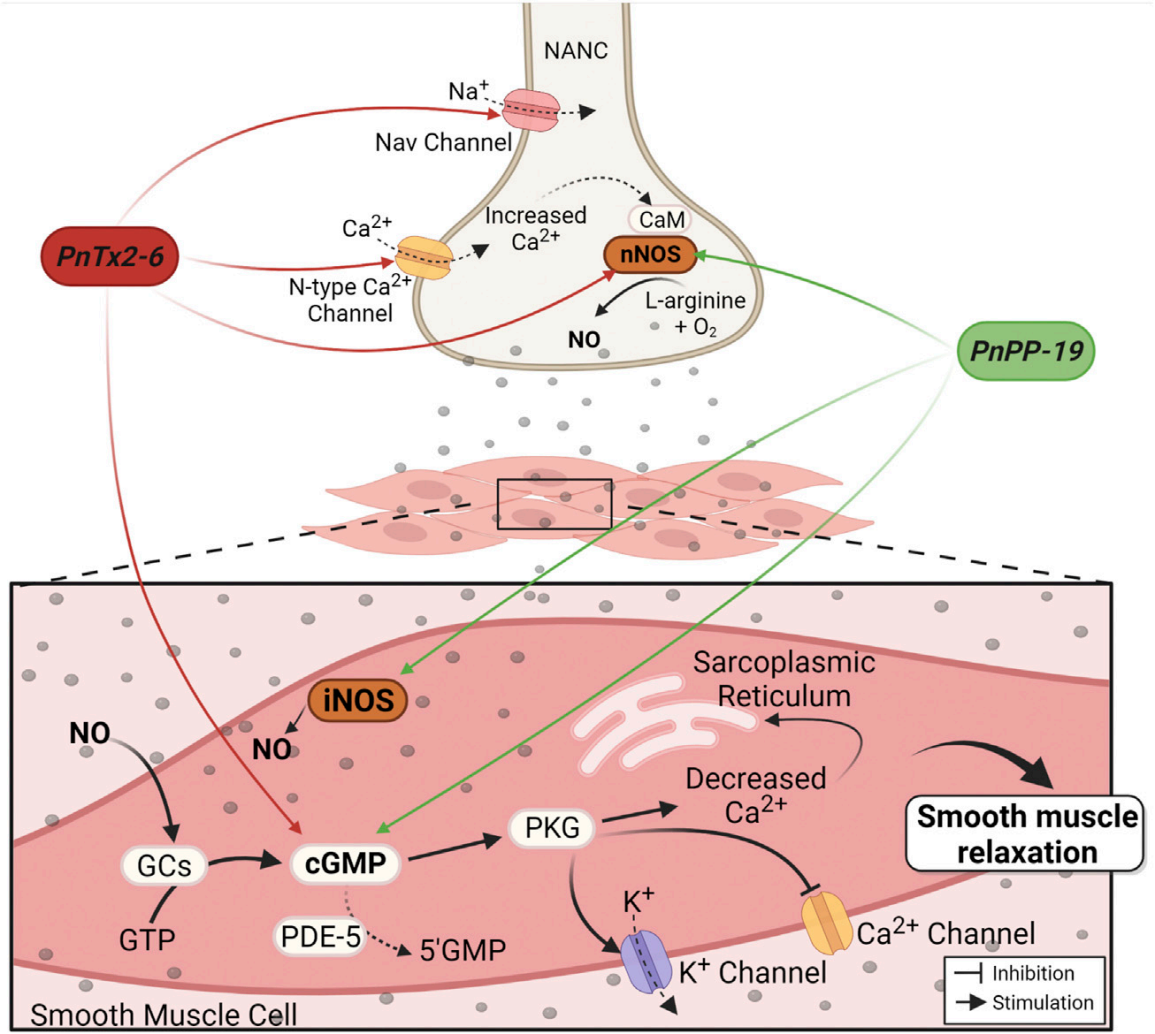
Mechanism of PnTx2-6 toxin and PnPP-19 peptide in the corpus cavernosum. Red arrows indicate PnTx2-6 actions and green arrows indicate PnPP-19 actions. The neuronal depolarization caused by PnTx2-6 (by delay Na^+^ channel inactivation current) leads to the opening of N-type Ca^2+^ channels, increasing its influx. Increase in intracellular Ca^2+^ triggers the NO production and availability. NO production stimulates guanylate cyclase enzyme (GCs), rising cGMP, which stimulates PKG resulting in blockade of Cavs channels and opening of Kvs channels. The aftermath is decreased Ca^2+^ inside the smooth muscle cell leading to relaxation and penile erection. Also, PnTx2-6 does not inhibit the action of the enzyme PDE-5. PnPP-19 (green arrows) increases NO and cGMP *via* nNOS and iNOS activation. PnPP-19 promotes CC relaxation without acting on voltage-dependent Na^+^ channels, neither N-type Ca^2+^ channels, and as PnTx2-6 it does not inhibit the action of PDE-5 or exerts an effect on Ach. Abbreviations: PKG, protein kinase G; GTP, guanosine triphosphate; nNOS, neuronal nitric oxide synthase; iNOS, induced nitric oxide synthase; CaM, calmodulin; NANC, nitrergic nerves; PDE-5, phosphodiesterase-5 enzyme; cGMP, cyclic guanosine monophosphate; NO, nitric oxide; GCs, soluble Guanylate cyclase. Created with BioRender.com.

The PnTx2-6 toxin potentiates erection in anesthetized rats through the release of NO into penile tissue (Nunes et al., 2008). This effect was inhibited by pretreatment with N(G)-nitro-L-arginine methyl ester (L-NAME), a non-selective inhibitor of NOS. Under electrical field stimulation (EFS), *corpus cavernosum* strips of normotensive rats pre-contracted with phenylephrine, presented greater relaxation in the presence of this toxin compared to the relaxation produced only by EFS (Nunes et al., 2010). The mechanism proposed suggests that, by delaying the inactivation of Na^+^ channels in nitrergic nerves, PnTx2-6 depolarizes the membrane, which leads to the opening of N-type Ca^2+^ channels, enhancing this ion influx (Figure 2). Increased intracellular Ca^2+^ stimulates signaling in the NO/cGMP pathway, boosting NO production and availability (Nunes et al., 2012b). This hypothesis is partially supported by previous data demonstrating the activity of this toxin in Na^+^ channels (Matavel et al., 2009). Additionally, blockade of the N-type Ca^2+^ channel with omega conotoxin GVIA, inhibits CC relaxation induced by PnTx2-6. This toxin restores erectile function in hypertensive rats (DOCA-salt) (Nunes et al., 2008) and improves erectile function in middle-aged rats (60–61 weeks) (Nunes et al., 2012a). Moreover, PnTx2-6 increases cavernosal relaxation in strips isolated from diabetic mice and eNOS knockout mice (Nunes et al., 2012b).

Interestingly, even when injected intracerebrally, PnTx2-6 induces penile erection (Yonamine et al., 2005; Soares et al., 2014). While the involvement of NO/cGMP pathway has been consistently demonstrated in many studies, the mechanisms by which this toxin would mediate penile erection are not completely elucidated and it might act not only systemically, but also centrally. Both, PhTx2 fraction and PnTx2-6 toxin can breakdown the blood brain barrier (BBB). The integrity of BBB was verified through the analysis of the expression of essential basement membrane structures and major proteins of endothelial caveolae (Soares et al., 2014; Silva et al., 2018). Radiolabeled PnTx2-6 subcutaneously injected in rats was observed in the brain, penis, and testicles. But only small amounts were observed in the brain and testicles compared to the penis (Nunes et al., 2010). Corroborating this data, iodinated PnTx2-6 injected intraperitoneally in mice penetrates the BBB reaching brain areas associated with sexual function, as demonstrated by an increased activation of c-fos, an indirect marker for neuronal activity, in the paraventricular and stria terminalis nucleus (Troncone et al., 2011). The major excitatory neurotransmitter in the brain, Glutamate, has a large array of normal physiological functions, including facilitating penile erection by acting in several areas, especially glutamatergic neurons in the hypothalamus. Activation of the paraventricular nucleus in the hypothalamus by excitatory amino acids triggers intracellular Ca^2+^ influx, which activates nNOS and releases NO from these neurons contributing to tumescence in the penis (Melis and Argiolas, 2011). It seems that PnTx2-6 is able to reach the CNS and affect glutamate release (Silva et al., 2018). Additionally, in rat brain cortical synaptosomes, PnTx2-6 acts on Na^+^ channels inducing a prolonged depolarization of synaptosomes and increasing Ca^2+^ influx, which in turn leads to the fusion of synaptic vesicles full of glutamate in the presynaptic membrane. While authors suggested that could be more than one mechanism involved in the L-glutamate release by PnTx2-6, such as the reversal transport of glutamate, this effect seems to totally rely on Na^+^ channels and it is partially dependent on Ca^2+^ influx, not only *via* N-type Ca^2+^ channels, but P/Q type as well.

The synthetic peptide PnPP-19 also activates NO/cGMP pathway (Figure 2). The use of a fluorescent marker for NO (DAF-FM DA) evidenced an increase in NO release following relaxation during EFS in rat CC strips treated with PnPP-19. Increased cGMP levels were also observed. Both effects were blocked by a non-selective NOS inhibitor (L-NAME) and partially reduced by 7-nitroindazole (7-NI), a specific inhibitor of the nNOS enzyme. However, contrasting results obtained with the toxin, the effect of Ca^2+^ influx *via* N-type Ca^2+^ channels on PnPP-19-mediated erection was irrelevant (Silva et al., 2021). Likewise, previous result observed with the toxin PnTx2-6 (Nunes et al., 2010), activation of muscarinic receptors in the endothelium is not involved in this peptide’s action. In both cases there was non-participation of acetylcholine (Ach) in the mechanism of action of these compounds, implying that although PnTx2-6 and PnPP-19 exhibit many differences, they also share similar characteristics (Silva et al., 2021). Table 1 summarizes a comparison between the PnTx2-6 and PnPP-19 peptide.

At the gene level, PnPP-19 changes the expression of N*os* mRNA levels in CC strips. There was a reduction in the expression of *eNos* and a significant increase in *iNos*, with no changes observed for the nNos isoform after treatment with PnPP-19. In addition, the levels of phosphorylation in a few regulatory sites of these enzymes were checked. Reduced phosphorylation at the nNOS inhibitory site (Ser 852) after treatment with PnPP-19 was observed, which potentially resulted in increased nNOS activity. In contrast, no changes were observed in the activation site Ser1177 and deactivation site Thr495 of eNOS. These findings are corroborated by using knockout mice for both eNOS and nNOS enzymes (Silva et al., 2021). Treatment with PnPP-19 not only strongly induced gene expression for *iNos* but also significantly elevated its expression following EFS. N6-(1-iminoethyl)-L-lysine dihydrochloride (L-NIL), a specific blocker of the iNOS enzyme, suppressed the effect of PnPP-19 on erectile function. The knockout mice for iNOS showed no relaxation in their cavernous strips, which was surprisingly significantly less than that observed for control strips (iNOS KO + vehicle) (Silva et al., 2021). This study highlights the importance of the iNOS enzyme in the erectogenic mechanism, which is corroborated by Hung et al. using cavernosal smooth muscle cell cultures (Hung et al., 1995).

PDE-5 inhibitors are widely used, however, these drugs are not promising for some patients, being potentially dangerous for those with cardiovascular disorders (Dhir et al., 2011). While the PnTx2-6 toxin potentiates erectile function *in vivo*, it alters some cardiovascular parameters such as derivative of the ventricular pressure (±dP/dt) and left ventricular end-systolic pressure (LVESP) in isolated rat heart in a concentration-dependent manner. It also delays Na^+^ currents inactivation in rat cardiomyocytes (Silva et al., 2015). These properties along with the toxins complexity, high toxicity and induction of hyperalgesia, among others, strongly limits it being used as a drug candidate for ED. Contrariwise, the synthetic peptide PnPP-19 did not show any effect on rat cardiomyocytes or isolated hearts, even at high concentrations. PnPP-19 displayed no signs of toxicity after intraperitoneal administration of 0.1 or 5 mg/kg in mouse and showed low immunogenicity, which is an excellent outcome considering these concentrations are much superior compared to the mean lethal dose of 0.79 µg/mouse (31.6 μg/kg) for PnTx2-6. Side effects such as vascular congestion, cell necrosis, edema, cytoplasmic vacuolization, or nuclear condensation in the liver, kidneys, brain, heart, lungs were no longer observed. Contrasting with PnTx2-6, this peptide was inactive in any of the tested sub-types of Na^+^ channels (Silva et al., 2015). It is noteworthy that PnTx2-6 was active in several subtypes of Na_v_s, which facilitates the understanding of its high toxicity and nociceptive effect, as the different subtypes of channels are spread throughout the body and may be implicated in these effects. On the other hand, PnPP-19 was not active in any of the Na_v_s tested (Silva et al., 2015). Furthermore, the peptide is not hyperalgesic as PnTx2-6, instead PnPP-19 induced antinociception (Da Fonseca Pacheco et al., 2016; Freitas et al., 2016) and this effect is discussed in the next section of this review.

The potentiation of erectile function triggered by PnPP-19 is not dependent on PDE5 inhibition, and the combination of PnPP-19, by topical application, with sildenafil (intravenously) increased the intracavernosal pressure/medium arterial pressure (ICP/MAP) ratio in diabetic animals. These results can be explained due to the unrelated mechanisms of action of each drug. Both PnPP-19 and sildenafil act on the NO/cGMP pathway, PnPP-19 increases NO production and consequently cGMP levels within the smooth muscle cells in the CC, while sildenafil prevents decreasing cGMP levels (Silva et al., 2019). In addition, PnPP-19 was able to reverse the ED phenotype in spontaneously hypertensive rats and diabetic mice and rats. In a model of type 1 STZ-induced diabetic mice, relaxation following PnPP-19 treatment in CC strips was 83% higher compared to untreated animals (Silva et al., 2019).

Biodistribution assays suggested that the peptide labeled with ^123^I-PnPP-19 has a tropism for the testicles and penis of mice over time, after both topical and intravenous administration. Tests performed with the injected toxin PnTx2-6 radioactively labeled with technetium-99 revealed an accumulation of radioactivity in the penis, suggesting the existence of receptors for both compounds in this organ (Nunes et al., 2010; Silva et al., 2019). Currently, topical formulations to treat ED is a strong tendency, as it allows the use of drugs comfortably with fewer systemic side effects (Foldvari et al., 1998; Elnaggar et al., 2011). PnPP-19 was able to permeate human (De Marco Almeida et al., 2018) and rat skin (Silva et al., 2019), remaining active after skin permeation in both STZ-induced diabetic and healthy animals. The physicochemical properties of PnPP-19, which is amphipathic, contain a high percentage of cationic residues, an isoelectric point of about 10.16, and a net positive charge of 3.9 at pH 7, resulting in favorable conditions for the skin permeation (De Marco Almeida et al., 2018). Therefore, transdermal administration of PnPP-19 can be an alternative to the oral route (Cevc and Vierl, 2010; Silva et al., 2019). However, although PnPP-19 is very active by topical application, its permeation is not so high (De Marco Almeida et al., 2018; Silva et al., 2019). Currently, new approaches are being developed by our group to improve the peptide permeability across the human skin.

Still, considerable research needs to be done, but this peptide showed many advantages compared with the native toxin and valuable results highlighting its potential as a therapeutic drug. Clinical trials are ongoing in Brazil. Figure 2 summarize the actions of PnTx2-6 and the synthetic peptide PnPP-19 leading to erection.

## Pain and the Antinociceptive Effect of PnPP-19

### Pain

The concept of pain was recently redefined by the International Association for the Study of Pain (IASP) as “An unpleasant sensory and emotional experience associated with, or resembling that associated with, actual or potential tissue damage” (https://www.iasp-pain.org/publications/iasp-news/iasp-announces-revised-definition-of-pain/, accessed in 23 November 2021). Pain can be classified into different types, including chronic pain, which can last much longer than its usual course of acute injury in any disease, condition or the pain that may recur for months or years (Raffaeli and Arnaudo, 2017). These authors discuss the need to recognize pain as a disease, as they justify: “considering the enormous global burden of this condition”. According to IASP, which considers that biological, psychological, and social factors are involved in pain, the correct term when measuring the dolorous sensation in animals is nociception. Accordingly, substances decreasing this sensation are called antinociceptives. Indeed, test in animals provides valuable information about the analgesic potential of a drug. Pain is a very serious public health problem worldwide with a tremendous socioeconomic impact not only because of costly treatment but also lower productivity of affected people (Phillips, 2009). Therefore, the search for novel potent and selective analgesic drugs without side effects is critical (Manglik et al., 2016).

### Phoneutria Venom as Nociceptive and Some of Their Toxins as Antinociceptive Agents

It is known that stinging by some arthropods, as scorpions or spiders, including *P. nigriventer* (i.e., Schenberg and Lima, 1971; Bucaretchi et al., 2000) is usually followed by intense pain. The apparent contradiction is that such venoms seem to have nociceptive and antinociceptive molecules and has been observed in the venom of *Phoneutria nigriventer* (for a review, please see Lauria et al., 2020). Several spider and scorpion toxins are described as powerful antinociceptive agents, mostly acting on ionic channels (for review, please see Vandendriessche et al., 2010; Klint et al., 2015; Deuis et al., 2017; Cardoso et al., 2021; Ricardo Carvalho et al., 2021), or in opioid and/or cannabinoid systems (i.e., Martin-Eauclaire et al., 2010; Da Fonseca Pacheco et al., 2016; Freitas et al., 2016). As described, Phoneutria’s venom (PV) has a plethora of active molecules acting in different targets, some of them yet unknown. In general, studies of hyperalgesia have been evaluated for the Phoneutria crude venom, but not for its isolated toxins. Marangoni et al. (1993) suggested that PV could activate tissue kallikrein generating kinins, as kallidin, leading to increased vascular permeability increase and pain. Other studies have indicated that this venom targets different systems causing nociception (Zanchet and Cury, 2003; Gewehr et al., 2013). Nociception triggered by PV involves both peripheral and central mechanisms. The peripheral activity seems to be mediated by bradykinin B2 receptors, serotonin 5-HT4 receptors, NMDA (N-methyl-D-aspartate), AMPA (α-amino-3-hydroxy-5-methyl-4-isoxazolepropionic acid), NK1 and NK2 receptors, as well as tetrodotoxin sensitive sodium channels (TTX-s Nav), also including TRPV1 channels, and acid-sensing ion channels (ASIC). The central component of pain, on the other hand, involves tachykinin, glutamate and CGPR (calcitonin gene-related peptide) receptors and inflammatory mediators (Lauria et al., 2020).

Although it is very relevant to understand the *in vivo* response to the crude venom, it is still unclear which specific molecules in this venom interact with targets involved in the nociception. Probably, a synergistic or additive effect of different molecules on PV occurs *in vivo* and the nociception overcomes the antinociceptive effect observed for some isolated toxins. The studies of PV and its toxins, including those active in nociception, are widely documented (see De Lima et al., 2016; Peigneur et al., 2018; Lauria et al., 2020; Ricardo Carvalho et al., 2021, Lauria et al., 2020). However, the most studied toxins, isolated from this venom, are those causing antinociception. For example, several toxins isolated from the fraction PhTx3 (Figure 1) showing strong antinociceptive effect, are studied by our colleagues M.V. Gomez and C. Castro-Junior (Faculdade Santa Casa de Belo Horizonte, Brazil), especially the toxin named Ph-α-1ß. This toxin interacts with different Ca^2+^ channels and shows efficacy in many models of nociception, such as in cancer, fibromyalgia, pancreatitis, postoperative pain, diabetic neuropathic pain, and orofacial pain (Da Silva Junior et al., 2020; Rita Pereira et al., 2020; Tonello et al., 2020; Aoki et al., 2021; Garcia Mendes et al., 2021). Another fraction from the venom of *P. nigriventer*, called PhTx4 (Figure 1) was described having several insect toxins (for review see De Lima et al., 2016; Peigneur et al., 2018) targeting mainly Na^+^ channels of insects (De Lima et al., 2002; De Lima et al., 2007). Interestingly, we showed these toxins also exhibit antinociceptive effects in rats, without apparent toxicity to mammals.

To date, one of them, PnTx4 (5-5) (or r-ctenitotoxin-Pn1a) (Oliveira et al., 2019), when subcutaneously injected in rat paw, showed antinociceptive effect in different pain models, as those induced by PGE, carrageenan and glutamate. It was suggested that such effects could be mediated by inhibition of the glutamatergic system. Also, this toxin blocks the NMDA-receptor in mice hippocampal neurons (De Figueiredo et al., 2001). This toxin was also cloned and heterologously expressed in *E. coli* (Paiva et al., 2016). The expressed toxin (rPnTx4 (5.5)) showed different affinity and mode of action on insect and mammalian Navs expressed in *Xenopus leavis* oocytes. The most remarkable effect was slowing down of sodium current on cockroach NavBg, although the toxin also inhibited, to a lesser degree, sodium current in several tested mammal Navs, i.e., Nav1.3, Nav1.6 and slightly Nav1.5, Nav1.4 and Nav1.2. From the same fraction, the toxin PnTx4 (6-1) (or δ-Ctenitoxin-Pn1a) demonstrated antinociceptive effect in models of inflammation, neuropathic and acute pain, suggesting its mechanism of action involves the cannabinoid system through CB1-receptor, and the opioid system, through µ and δ-receptors (Emerich et al., 2016). Furthermore, a small engineered peptide, with 13 amino acid residues (PnAn13) (Emerich et al., 2020), from the sequence of the toxin (PnTx4 (6-1) shows antinociceptive effect in a rodent pain model, both centrally and peripherally. The mechanism of PnAn13 seems similar to that of the toxin, i.e., involving the cannabinoid and opioid systems. For further details about these active molecules on nociception, see a recent review (Lauria et al., 2020).

### The Antinociceptive Effect of PnPP-19

**FIGURE 3:**
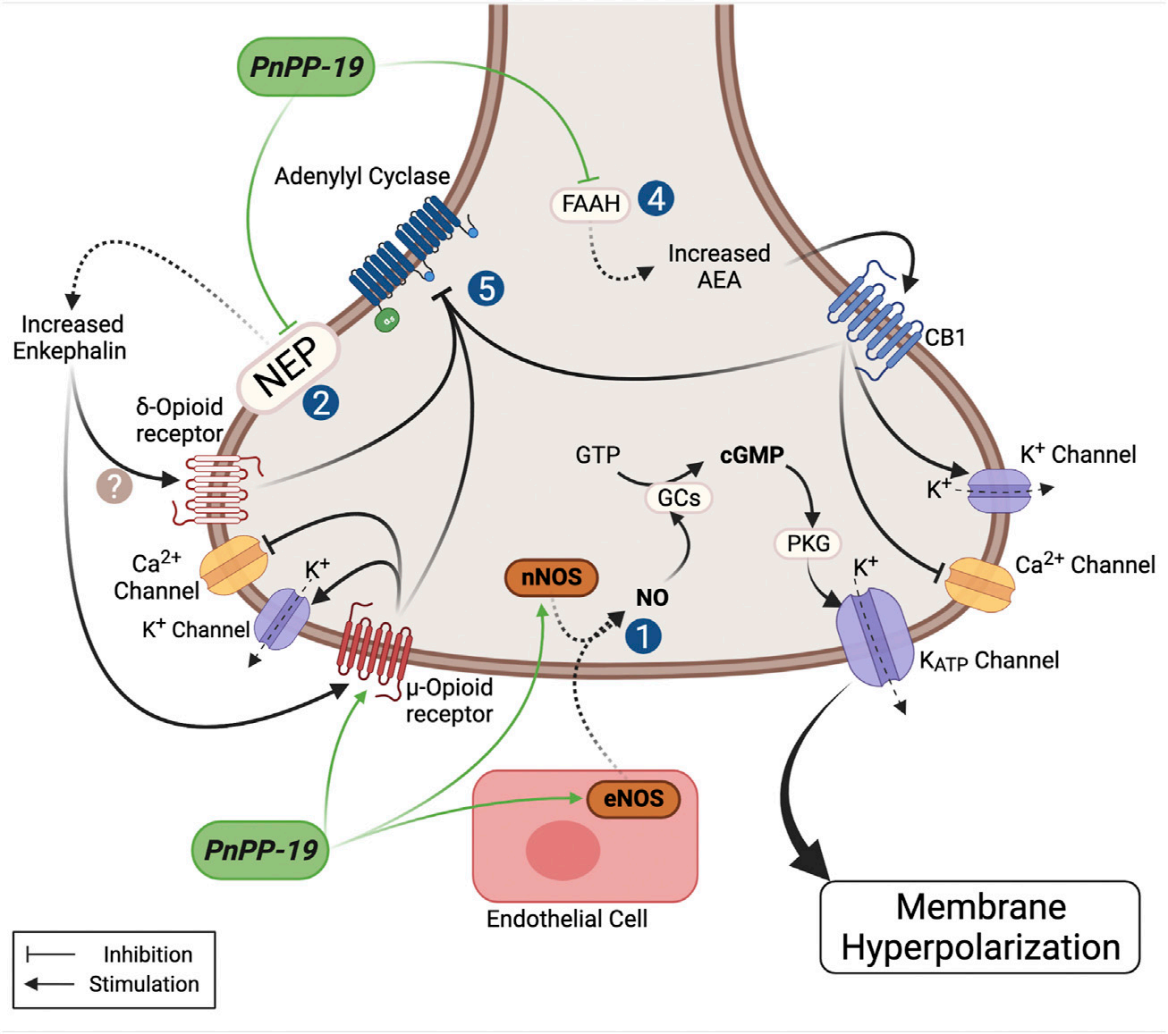
Schematic model showing the possible actions of the PnPP-19 peptide on antinociception. PnPP-19 acts peripherally and centrally, *via* the opioid and cannabinoid systems, involving the µ- and δ-opioid receptors and cannabinoid 1 -receptor. It was proposed that the concomitant activation of the pathway of opioids and cannabinoids may also be facilitated by heterodimerization between receptors of these two ways. The activation of opioid and cannabinoid receptors causes the inhibition of adenylate cyclase activity. PnPP-19 appears to act directly on the µ-, but not on the δ-receptors, transfected into Xenopus oocytes. Activation of CB1 receptors is likely to be caused by increased levels of anandamide, as inhibition of anandamide degradation has been shown to potentiate the effect of the peptide. The peptide inhibited the enzyme neprilisin (NEP or enkephalinase - EC 3.4.24.11) *in vitro*, which degrades enkephalin, and was hydrolyzed by this enzyme after 24h of incubation. The peptide also stimulated eNOs increasing NO, which *via* PKG leads to opening of KATP, resulting in hyperpolarization. PnPP-19 inhibited the calcium current in dorsal ganglion root cells, which was reversed by noloxane, an opiate blocker, suggesting that this effect must be *via* the opioid receptor, which leads to calcium channel blockade and opening of potassium channels. The antinociceptive action of the peptide *via* the CNS using blockers also have shown its action on the opioid and cannabinoid system. This whole signaling cascade, on the action of PnPP-19, leads to a decrease of neuronal excitability and blocking the release of neurotransmitters, reducing pain. For more details see text. NEP, neprilysin enzyme (neutral endopeptidase); nNOS, neuronal nitric oxide synthase; eNOS, endothelial nitric oxide synthase; GCs, soluble guanylate cyclase; PKG, phosphoprotein kinase G; GTP, guanosine triphosphate; FAAH, fatty acid amide hydrolase; AEA, N-arachidonoylethanolamine or anandamide. Created with BioRender.com.

PnTx2-6 toxin clearly induced hyperalgesia (Nunes, 2008) and although the mechanism involved in this activity was not investigated, we can suggest it may result, at least in part, from the action of this toxin on sodium channels (Matavel et al., 2009; Silva et al., 2015). Contrariwise, when examined, the peptide PnPP-19 showed peripheral and central antinociceptive effect (Da Fonseca Pacheco et al., 2016; Freitas et al., 2016) and as before described, this peptide does not have any activity on Navs. In rats injected with the potent proinflammatory mediator Prostaglandin E_2_ (PGE_2_), which is an acceptable model for producing severe hyperalgesia, local administration of PnPP-19 in the right hind paw produced a peripheral antinociceptive effect in a dose dependent manner (Freitas et al., 2016). Our studies suggested that antinociception induced by this peptide seems to involve the inhibition of neutral endopeptidase, neprilysin (NEP) (Freitas et al., 2016). In behavioral experiments, we observed that PnPP-19 also acts on the activation of CB_1_ cannabinoid, δ and µ opioids receptors. NEP is an enzyme responsible for the cleavage of many endogenous peptides, including the opioid encephalin, which regulates nociception in the body (Roques et al., 1993). It has been shown that increasing the levels of enkephalins by inhibiting their two inactivating ectopeptidases, neprilysin and aminopeptidase N, has analgesic effects in various models of inflammatory and neuropathic pain (for a review see Roques et al., 2012). Neprilysin is also one of the various enzymes that degrade bradykinin (Bujak-Gizycka et al., 2011). It is stated that bradykinin (Bk) shows pronociceptive and antinociceptive activity in a dose dependent manner (Aykan et al., 2019). Although it was suggested that Bk may be involved in the nociceptive activity of PV (see above) we did not investigate if Bk could have some role in the activity of PnPP-19. It seems unlikely, because kinins could alter the pression and the medium arterial pressure (MAP), measured alongside the experiments of erectile function in rats, did not variate significantly. On the other hand, PnPP-19 was also tested in aorta rings and no relaxation effect was observed in this arteria (not shown). Interestingly, patients presenting ED showed down-regulation of opiorphin, an endogenous inhibitor of NEP in humans (Tong et al., 2008), which presents some similarities with the PnPP-19 sequence. The nociceptive threshold to thermal stimulation was evaluated after PnPP-19 intracerebroventricular administration in mice at different doses in the presence or absence of antagonists for CB_1_ and opioid receptors. In the CNS, PnPP-19 showed, as observed peripherally, activation of µ- δ-opioid and CB_1_ cannabinoid receptors associated to antinociception *via* (Da Fonseca Pacheco et al., 2016). At this point, the aspects already known of the PnPP-19 mechanism in pain is summarized in Figure 3, although further investigations are needed to clearly state its mode of action.

The paraventricular nucleus of the hypothalamus contains CB_1_ receptors, and it modulates erection and sexual behavior. The mechanism suggested to explain this effect is that local antagonism of cannabinoid receptor CB_1_ results in decreased GABA release (major neurotransmitter inhibitor in the brain) and augmented glutamate, which causes an increased NO production and oxytocin release (Argiolas and Melis, 2004; Lovera et al., 2007). Oxytocin is an established contributor to erections, sexual arousal, and ejaculation (Veening et al., 2015).

In another study, the direct activation of different subtypes of opioids receptors by PnPP-19 was investigated. In oocytes µ, δ and k human opioids receptors were co-expressed with GIRK1/GIRK2 and RGS4. It was shown that PnPP-19 selectively activates with low potency, only μ-opioids receptors, but not δ- or k-opioids receptors, suggesting that activation of δ-subtype receptor *in vivo* may occur *via* indirect pathway (Freitas et al., 2018). PnPP-19 also inhibited Ca^2+^ evoked current in dorsal root ganglion neurons (DRGs) with an efficacy comparable to morphine, and this effect was inhibited by an unspecific opioid antagonist, naloxone, suggesting that the peptide may be acting *via* opioid receptor (Freitas et al., 2016). Although the benefits of opioid drugs as analgesics (i.e. morphine, fentanyl and oxymorphone) are widely recognized, these substances cause several side effects, such as respiratory paralysis (Wilson and Saukkonen, 2004; Benyamin et al., 2008), and most of these effects seem to be related to the recruitment of the β-arrestin pathway following the activation of μ-opioids receptors. This recruitment was not observed in HEK293T cells, co-expressing μ-opioids receptor-Yc and beta-arrestin2-Yn, stimulated by different concentrations of PnPP-19 (Freitas et al., 2018), which highlights another positive feature to this peptide as a possible drug to treat pain.

The participation of the opioid system was previously highlighted as part of the action mechanism of some scorpion neurotoxins that induce antinociception (Martin-Eauclaire et al., 2010), which supports the analgesic role of PnPP-19.

NO is not only crucial for erectile function, but it is also described to have antinociceptive effect. It is well accepted that many endogenous and exogenous substances induce antinociception due to activation of the nitrergic system. The intraplantar administration of NO donor, sodium nitroprusside, induces antinociception in rat’s paw made hyperalgesic with PGE_2_, and this effect is blocked in the presence of an inhibitor for guanylate cyclase (Durate et al., 1990). NO has a remarkable role in the nociceptive pathway (Cury et al., 2011) and it is part of the mechanism of action of some peptide toxins (Gazerani and Cairns, 2014). Accordingly, the involvement of NO/cGMP pathway in the peripheral antinociceptive effect induced by PnPP-19 was evaluated and confirmed. In addition, results showed that this effect is mediated by ATP-sensitive potassium channels (K_ATP_). In the NO/cGMP pathway, PKG might be activated, stimulating the opening of K_ATP_ channels. Administration of a specific cGMP inhibitor (OQD) and selective K_ATP_ channels blocker (Glibenclamide) partially inhibited the antinociceptive effect induced by PnPP-19 (Freitas et al., 2017). Altogether, it seems that this peptide has a widespread antinociceptive action and it is a feasible compound for developing a novel drug. Of note, it is crucial to consider that NO has a dual effect, and this gas may positively mediate pain in both systems, central and peripheral (For review, please see: Cury et al., 2011). Still, many experiments need to be performed to elucidate the antinociceptive effect of this peptide and clarify other pathways possible involved.

## Hypotensive and Neuroprotective Effects of PnPP-19 in the Eyes

### Glaucoma

Glaucoma is one of the main disease responsible of blindness worldwide and also a major public health problem (World Health Organization, 2021). Nowadays, about 80 millions individuals are affected by this disease, especially people at a very advanced age, which might be associated with increase of oxidative/inflammatory components and aging (Shih and Calkins, 2012; Flaxman et al., 2017). This disorder belongs to a heterogeneous group of eye diseases characterized by chronic loss of retinal ganglion cells (RGCs). In the retina, their axons merge to form the optic nerve, responsible for transferring visual neural signals from the eye to the brain. RGCs are extremely sensitive and suffer progressive apoptotic death after ischemia caused by vasoconstriction or raised intraocular pressure (Shih and Calkins, 2012; Kastner and King, 2020).

Based on the morphology of the anterior chamber, glaucoma can be named as open- or closed-angle and as primary or secondary. The angle refers to the drainage angle between the iris and the cornea that eventually can be obstructed and blocks the flow of aqueous humor, leading to increase of intraocular pressure (IOP) (Lusthaus and Goldberg, 2019). Primary glaucoma is the most common category and occurs in 74% of cases (Kapetanakis et al., 2016). This type of glaucoma is associated to increased pressure over the years, thus it is diagnosed when the damage is already very advanced (Lusthaus and Goldberg, 2019). Secondary glaucoma is characterized by increase IOP because of ocular or systemic disorders. As an example of this, in the retrospective study performed by Hoeksema and collaborators (Hoeksema et al., 2017), the authors researched ocular complications most commonly seen during follow-up for uveitis. The results revealed that’s 75% of the patients had elevated IOP, and 15% developed glaucoma.

Glaucoma treatment is focused on the reduction of IOP once all patients with glaucoma may benefit from adequate IOP values. Nowadays, topical hypotensive ocular medicines continue to be the main option in glaucoma therapy (Lusthaus and Goldberg, 2019). However, some patients with glaucoma remain losing their RGCs despite IOP reduction. In this scenario, searching for molecules capable of protecting these cells has intensified (Mahmoud et al., 2019). Among IOP-lowering drugs, brimonidine has been extensively studied, due to its potential to reduce the IOP, and its neuroprotective effect on retinal cells. Although its use is limited by high rates of ocular allergy, hyperemia, and discomfort (Shih and Calkins, 2012; Oh et al., 2019).

In this context, NO has gained attention as new target for the treatment of glaucoma. In the eye, NO/GMPc pathway could be involved in homeostatic processes, such as regulation of aqueous humor dynamics, retinal neurotransmission, and phototransduction (Cavet et al., 2014). Therefore, changes in NO synthesis could lead to many ocular diseases as glaucoma and retinal degeneration (Drago and Bucolo, 2010). NO is produced in the anterior and posterior ocular segments, a fact evidenced by the presence of NOS isoforms in these tissues. The nNOS is found in nerve fibers in the cornea, limbus, and lens epithelium whereas eNOS is seen in the ciliary muscle, Schlemm’s canal, and in the trabecular meshwork. NO synthesis is mediated by iNOS. These tissues are related to the IOP control (Schneemann et al., 2003; Stamer et al., 2011; Cavet et al., 2014; Chang et al., 2015; Tsai et al., 2017). Schlemm’s canal, a vein-like structure, and the trabecular meshwork are highly contractile. So, the endogenous production and release of NO promotes relaxation of vascular tone and juxtacanalicular trabeculate which can facilitate the outflow of aqueous humor and, therefore, induces the IOP reduction (Wiederholt et al., 1994; Schneemann et al., 2002; Goel et al., 2010). On the other hand, NO produced by eNOS isoform can induce a protective response after ischemic episodes. The constitutive release of NO is reduced after the onset of ischemia. Thus, therapies capable of promoting an increase in local availability of NO during this critical period could improve blood flow by vasodilating and, consequently, acting as a neuroprotective agent against retinal ischemia (Kaur et al., 2006; Toda and Nakanishi-Toda, 2007). Accordingly, novel NO donors are needed to promote the IOP reduction, and protects retinal cells of ischemic injury (Schuman et al., 1994; Drago and Bucolo, 2010). Considering all this information in addition to our previous results showing PnPP-19 as an NO inducer, we have tested this peptide as a possible drug able to reduce IOP.

### Effects of PnPP-19 in Eyes

Aiming to verify potential ocular use of PnPP-19, the irritant potential of this peptide was evaluated in the eye by using the Hen’s egg test in the chorioallantoic membrane (HET-CAM), and the ocular irritation index was calculated (Silva et al., 2020). Only the positive control was classified as severely irritating, the negative control and the different concentrations of PnPP-19 tested showed no signs of toxicity. This test is widely used to assess the irritant potential of products intended for topical use such as emulsions (Ali et al., 2012), gels (M.a. Fathalla et al., 2017), and various cosmetic preparations (Steiling et al., 1999; Derouiche and Abdennour, 2017).

Considering the absence of toxicity in the HET-CAM test, the safety for topical use of this drug *in vivo* was investigated using electroretinography (ERG) test. The ERG is a non-invasive test capable of detecting small areas of retinal dysfunction (Lai et al., 2007). No difference was seen in the pattern of ERG curves for eyes treated with PnPP-19 after 1, 7, and 15 days. Also, histological analysis showed no morphological changes (Silva et al., 2019).

**FIGURE 4:**
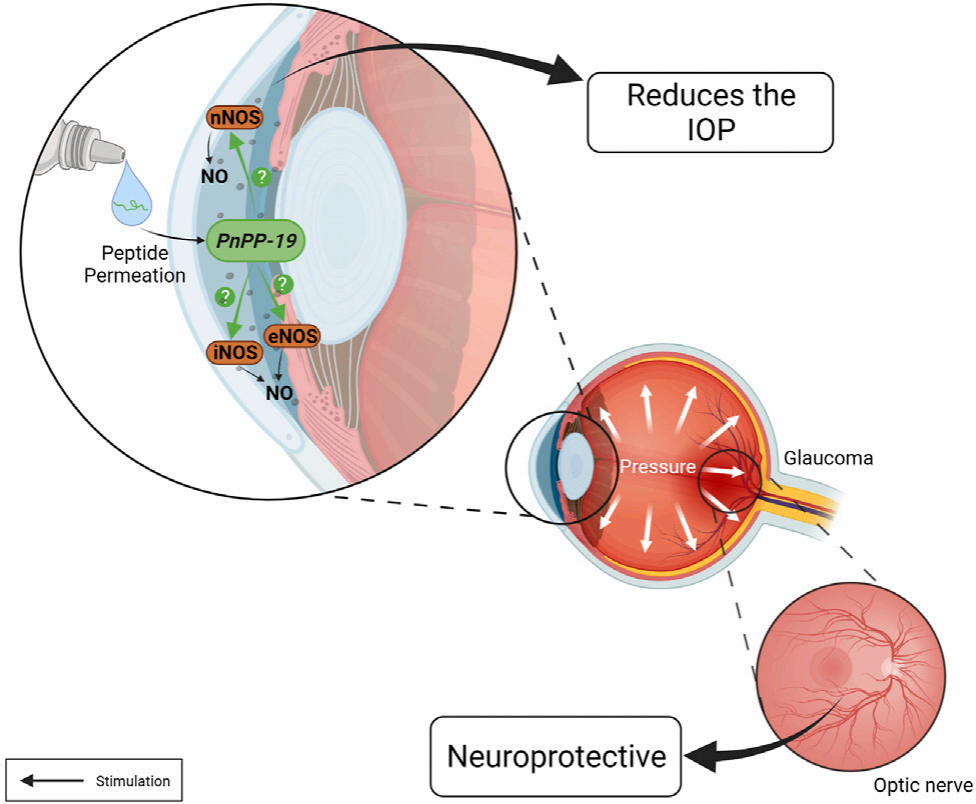
Actions of the PnPP-19 peptide in the eye. PnPP-19 a NO-inducer peptide, when topically applied as eye drops, reduces IOP after a single instillation, both in healthy and hypertensive eyes, without eliciting apparent toxicity. However, it is unknown which NOS is activated by PnPP-19. This peptide may promote neuroprotection by preventing the progression of optic nerve degeneration. Created with BioRender.com.

PnPP-19 as eyedrop (80 µg/20 µl) was able to permeate the cornea and reduce the IOP of normotensive rats. This effect lasted approximately 24 h after a single drop of the compound and the effect was shown to be induced by NO release. The ability of PnPP-19 to decrease IOP was also evaluated in an experimental model of ocular hypertension induced by intraocular injection of hyaluronic acid based on Moreno et al., 2005; Franca et al., 2014. The results revealed that, once ocular hypertension was established, the group treated with a single dose of PnPP-19 (80 µg/20 µl) significantly reduced IOP, compared to control group that received only vehicle (saline). Furthermore, even after 24 h, the IOP remained statistically reduced for groups treated with peptide compared with the vehicle group. The PnPP-19 treated group also had lower IOP than the group treated with Bimatoprost, a commercially available antiglaucoma drug (Silva et al., 2019). Histological analysis showed significant loss of RGC in eyes of rat treated with vehicle. However, in the group treated with PnPP-19, the number of RGC was similar to the healthy eyes, suggesting that treatment with PnPP-19 was able to protect these cells (Silva et al., 2020) (Figure 4).

Although there is a wide availability of anti-glaucoma drugs, still there is an important medical need in this field (Shalaby et al., 2020). Currently, most drugs for the treatment of glaucoma act by reducing the flow of aqueous humor or increasing its output through the uveoscleral route. However, the tissues compromised in glaucoma responsible for increasing IOP remains an important problem to be addressed, although little explored (Weinreb et al., 2014). In this context, the peptide PnPP-19 appears as an interesting therapeutic option.

## Conclusion

Some venoms contain large amounts of peptide toxins, and these molecules show a wide range of pharmacological activities of great value for better understanding several biological processes. Over the last 3 decades, significant scientific advances increased our knowledge regarding *P. nigriventer* spider venom, which has many peptide toxins, including PnTx2-6, a very toxic molecule, highlighted in this review. PnTx2-6 proves to be an excellent pharmacological tool for studying erectile function. It enhances penile erection in normotensive rats and restores erectile function in hypertensive, elderly rats, and diabetic mice *via* NO/cGMP activation. However, because PnTx2-6 is highly toxic and induces nociception, its therapeutic use is impossible. Seeking to overcome such limitations, the synthetic peptide PnPP-19 was proposed. It is easily synthesized, does not have disulfide bridges, is stable, and it potentiates murine erectile function, inducing irrelevant immunogenicity and no evident signs of toxicity (Silva et al., 2015). Surprisingly, PnPP-19 did not act on Na_v_ channels as its toxin precursor, and does not affect the heart of rats, but it acted by modulating the expression of NOS enzymes in CC (Silva et al., 2015, 2021). The mode of action of PnPP-19 is different in relation to the current pharmacotherapy since it increases the production of NO/cGMP and has a synergistic, or additive effect with PDE-5 inhibitors. To confirm if this effect is synergistic or additive, a more detailed study, as involving for example involving isobolographic analysis, should be done. PnPP-19 was able to cross the skin, which allows its use in a formulation for topical application, and it reversed the erectile dysfunction phenotype presented by hypertensive and diabetic animals. However, the permeation was limited, but it still showed good efficacy (De Marco Almeida et al., 2018; Silva et al., 2019). To improve the permeation, we are trying to develop other formulations and minimize the costs. In contrast to the toxin, the peptide was not hyperalgesic, showed peripheral, and central antinociceptive effect (Da Fonseca Pacheco et al., 2016; Freitas et al., 2016). This probably is due to the inhibition of neutral endopeptidase (as shown *in vitro*), which prevents the destruction of endogenous opioids, besides acting on the activation of CB1 cannabinoid, δ and µ - opioids receptors, as pharmacologically verified. PnPP-19 also inhibited Ca^2+^ evoked current in DRGs, probably *via* opioid receptor, with an efficacy comparable to morphine. Supporting this hypothesis, it was shown that this peptide binds with specificity to the µ - opioid receptor (Freitas et al., 2016). The induced peripheral antinociceptive effect by the peptide was shown to involve NO/cGMP pathway (Freitas et al., 2017). As a NO-inducer peptide, PnPP-19 was able to reduce IOP in rats healthy or with ocular hypertension for 24 h after a single dose (eye drop), without toxicity. Besides, it promoted neuroprotection by preventing the progression of optic nerve degeneration (Silva et al., 2020). These results highlight its potential as a drug for the treatment of glaucoma and other neurodegenerative eye diseases. The data presented here illustrate the importance of studies with animal toxins as they enabled the design of PnPP-19, a versatile and promising molecule to treat ED, nociception, and glaucoma. So, alongside the immense pharmacological potential of the natural molecules from animal venoms, they also represent a source of inspiration to build other useful and non-toxic molecules with clinical and biotechnological applications.
